# The Role of Alcohol, LPS Toxicity, and ALDH2 in Dental Bony Defects

**DOI:** 10.3390/biom11050651

**Published:** 2021-04-28

**Authors:** Hsiao-Cheng Tsai, Che-Hong Chen, Daria Mochly-Rosen, Yi-Chen Ethan Li, Min-Huey Chen

**Affiliations:** 1 Graduate Institute of Clinical Dentistry, School of Dentistry, National Taiwan University, Taipei 100, Taiwan; huangmimi1101@gmail.com; 2 Department of Dentistry, National Taiwan University Hospital, Taipei 100, Taiwan; 3 Department of Chemical and Systems Biology, Stanford University, School of Medicine, Stanford, CA 94305, USA; chehong@stanford.edu (C.-H.C.); mochly@stanford.edu (D.M.-R.); 4 Department of Chemical Engineering, Feng Chia University, Taichung 407, Taiwan

**Keywords:** ALDH2, dental bone loss, LPS, alcohol, periodontal disease, osteoblast, mineralization

## Abstract

It is estimated that 560 million people carry an East Asian-specific ALDH2*2 dominant-negative mutation which leads to enzyme inactivation. This common ALDH2 polymorphism has a significant association with osteoporosis. We hypothesized that the ALDH2*2 mutation in conjunction with periodontal *Porphyromonas gingivalis* bacterial infection and alcohol drinking had an inhibitory effect on osteoblasts and bone regeneration. We examined the prospective association of ALDH2 activity with the proliferation and mineralization potential of human osteoblasts in vitro. The ALDH2 knockdown experiments showed that the ALDH2 knockdown osteoblasts lost their proliferation and mineralization capability. To mimic dental bacterial infection, we compared the dental bony defects in wild-type mice and ALDH2*2 knockin mice after injection with purified lipopolysaccharides (LPS), derived from *P. gingivalis* which is a bacterial species known to cause periodontitis. Micro-computed tomography (micro-CT) scan results indicated that bone regeneration was significantly affected in the ALDH2*2 knockin mice with about 20% more dental bony defects after LPS injection than the wild-type mice. Moreover, the ALDH2*2 knockin mutant mice had decreased osteoblast growth and more dental bone loss in the upper left jaw region after LPS injection. In conclusion, these results indicated that the ALDH2*2 mutation with alcohol drinking and chronic exposure to dental bacterial-derived toxin increased the risk of dental bone loss.

## 1. Introduction

Approximately 10–15% of adults suffer from periodontal disease with dental bone loss leading to tooth loss in patients and affecting their quality of life [1,2]. Published studies have also indicated that periodontal disease can induce systemic complications [2,3]. For example, an oral bacterium, *Porphyromonas gingivalis*, induces chronic infection of gingiva and tooth loss, and also enhances the risk of cardiovascular diseases such as heart attack [4]. Additionally, people with periodontal disease also have a three-fold increased risk of diabetes [5]. One possible mechanism of this disease is that the microbials in periodontal tissues may induce the secretion of inflammatory factors such as TGF-α. These factors interfere with the regulation of a host’s immune response by inhibiting the functions of the receptors and mediators of these factors, and therefore lead to diabetes [6]. Other studies have also confirmed that periodontal disease can increase the risk of the rheumatoid arthritis, metabolic disease, and adverse pregnancy outcomes [7,8]. Therefore, understanding the mechanism of dental bone loss in periodontal disease is important for reducing the risks of systemic complications.

There have been several reported causes of dental bone loss in periodontal disease which increase the risk of congenital disease. For example, hormone or endocrine disorders might inhibit the repair of collagen fiber in the gingival tissue and cause alveolar bone loss [9]. Unhealthy lifestyle factors, such as smoking, poor oral hygiene, and alcohol use, also increase the risk of periodontal disease and dental bone loss. Smoking promotes the accumulation of dental microorganisms and increases the risk of dental bone loss by several fold in smokers as compared with non-smokers [10]. Alcohol drinkers are also more prone to having dental bone loss than non-drinkers [11]. Chronic alcohol consumption is known to affect the immune system and increase periodontal inflammation which is likely the mechanism that leads to dental bone loss.

The role of genetic factors in alcohol-induced dental bone loss has not been explored thus far. The polymorphism of a single amino acid substitution which leads to the loss of enzyme function in the mitochondrial aldehyde dehydrogenase 2 (ALDH2) gene is well known [12]. Following alcohol consumption, people with ALDH2*2 mutation cannot metabolize acetaldehyde efficiently which leads to elevated acetaldehyde in the saliva, bloodstream, and the well-characterized tachycardia and facial flushing reaction [13,14]. The ALDH2*2 mutation is common in the East Asian population. It is estimated that 560 million East Asians are affected by the ALDH2*2 mutation [15]. Acetaldehyde toxicity has been reported to have a negative effect on bone growth [16]. A previous study showed that the risk of dental bone loss in periodontal disease was elevated with the consumption of >60 g of alcohol per day [17]. Nishida et al. also showed a positive relationship among ALDH2 genotype, alcohol consumption, and a smoking habit with the risk of dental bone loss in patients with periodontal disease [18]. It has been shown that heavy alcohol consumption was highly correlated with increased occurrence of dental bone loss in periodontal disease. However, the mechanism linking ALDH2 deficiency and bone loss in periodontitis is still not clear. The purpose of this study is to understand the roles of ALDH2, and the effects of ALDH2*2 mutation, alcohol drinking, and bacterial infection on dental bone loss. To accomplish this, in vitro and in vivo animal studies were both performed. We used an animal model of ALDH2*2, and lipopolysaccharides (LPS), known lipoglycans and endotoxins from the oral bacterium, *Porphyromonas gingivalis*, were injected to create inflammation. In addition, alcohol administration through oral exposure of 10% alcohol in drinking water was provided to observe the effects of dental bony defects in mice. In this study, we demonstrated that under LPS-induced toxicity and alcohol exposure, deficiency in the ALDH2 enzyme played a significant role in dental bone loss. The results from this study may provide important clinical information and a potential therapeutic target for the treatment or prevention of dental bone loss in periodontal disease, especially for the large population of East Asians carrying the vulnerable ALDH2*2 genotype.

## 2. Materials and Methods

### 2.1. Gene Sequence

The shRNA sequence of ALDH2 was obtained from the RNAi database (Clone name NM_000690.2-981s1c1, Academia Sinica, Taipei, Taiwan). The ALDH2 shRNA vector was purchased from Academia Sinica. Osteoblasts expressing silenced human ALDH2 were developed by transfecting cells with lentivirus carrying the ALDH2 shRNA. The targeted ALDH2 sequence was GCAGATCATTCCGTGGAATTT.

### 2.2. Cell Lines, Cell Culture, and Transfection Conditions

The human osteoblast (transfected with SV40 large T antigen) cell line (hFOB 1.19) was obtained from Bioresource Collection and Research Center (BCRC, Hsinchu, Taiwan). Cells were cultured at 37 °C in a humidified incubator with 5% CO_2_. Dulbecco’s modified Eagle’s medium containing 10% fetal bovine serum (FBS), 100 unit/mL penicillin +100 μg/mL streptomycin (Biological Industries, Cromwell, CT, USA), and 2 mM L-glutamine (Gibco, Thermo Fisher Scientific Inc., Vista, CA, USA) were used for culture. Cells were grown to 80% confluence, and then passaged. Osteoblasts expressing silenced human ALDH2 were selected by transfecting cells with lentivirus (Invitrogen, Thermo Fisher Scientific Inc., Vista, CA, USA) carrying the ALDH2 shRNA (multiplicity of infection (MOI) = 10) vector. The MOI is defined as the ratio of infectious agents to infection targets. It is also the ratio of viral particles to the number of target cells in a defined space, such as in a cell culture well. The cell line, designated as ALDH2 knockdown osteoblasts were confirmed with low ALDH2 expression by Western blot analysis using the ALDH2 antibody (rabbit monoclonal antibody (EPR4493), Abcam, Cambridge, MA, USA). Osteoblasts transfected with an empty lentivirus were used as the control. Cells at 3–4 passages were used for the MTT assay.

### 2.3. MTT Assay

Cell viabilities of both control and ALDH2 knockdown transfected osteoblasts were analyzed by using the assay of 3-(4,5-dimethylthiazol-2-yl)-2,5-diphenyl tetrazolium bromide (MTT compound, Sigma-Aldrich, St. Louis, MO, USA) [19]. Both passaged control and ALDH2 knockdown transfected osteoblasts were seeded at a density of 1 × 10^4^ cells per well in a 96-well microplate (*n* = 8) in the DMEM culture medium for 7 and 14 days. On Day 7 and Day 14, MTT was added to each culture well at a final concentration of 2 mg/mL followed by an incubation at 37 °C for 4 h. Then, the medium was harvested and centrifuged at 900 rpm for 5 min and aspirated. The pelleted formazan reaction products were dissolved in 200 μL of dimehtyl sulfoxide (DMSO, Sigma-Aldrich) solution and shaken for 15 min. The optical density of the formazan solution was recorded using an enzyme-linked immunosorbent assay (ELISA) plate reader (ELx 800, BioTek Instruments Inc., Winooski, VT, USA) at 570 nm. The absorbance was proportional to the metabolic activities and viabilities of the cells.

### 2.4. Mineralization Assay by the Measurement of Alizarin Red S

In this assay [19], both control and ALDH2 knockdown transfected osteoblasts were cultured in the induction medium supplemented with 100 nM dexamethasone, 10 mM β-glycerophosphate, and 50 mg/mL ascorbic acid (Sigma-Aldrich) at two different seeding densities (low, 5 × 10^3^ cells/mL and high, 1 × 10^5^ cells/mL). The degree of mineralization was observed by phase contrast microscopy from the independent culture wells of the control and ALDH2 knockdown groups.

### 2.5. In Vivo Study

The animal use was approved and followed the guidelines of the Laboratory Animal Center at the National Taiwan University College of Medicine, Taiwan. The ALDH2*2 knockin mice representing the ALDH2*2 single amino acid substitution of human ALDH2*2 were generated and obtained from Prof. Daria Mochly-Rosen’s lab, Stanford University. The construction, characterization, and phenotype of the ALDH2*2 mice were as described previously [20]. Four C57BL6 ALDH2 wild-type male mice (6 weeks old) were used as the control group, and four age-matched C57BL6 ALDH2*2 knockin male mice (6 weeks old) were used as the experimental group. The left upper 1st molar and 2nd molar interdental alveolar bone and between the 2nd and 3rd molar interdental areas of all mice were injected with 2 μL (20 μg) [21] of purified lipopolysaccharide from *Porphyromonas gingivalis* (p-gingivalis LPS, InvivoGen, San Diego, CA, USA) under anesthesia and manipulated under a microscopy. The injection was performed twice a week and continued for 6 weeks. The corresponding right side of the upper 1st molar and 2nd molar interdental alveolar bone areas and between the 2nd and 3rd molar interdental areas of all mice were injected with 2 μL vehicle (endotoxin-free water), twice a week, and continued for 6 weeks as the control. Drinking water with 10% alcohol and standard dry food were administered to all mice. After 6 weeks, all mice were sacrificed for evaluation by micro-CT and histological observation with hematoxylin and eosin staining.

### 2.6. Evaluation by Micro-Computed Tomography (Micro-CT) Imaging

Micro-CT imaging protocol was as described in our previous study [22]. To obtain micro-CT images, upon animal sacrifice, samples of upper jaws of all mice were collected, trimmed, and fixed in 10% formalin solution for at least three days. Micro-CT images were captured through a micro-CT scanner (SkyScan1176, Bruker Corp., Konitch, Belgium). The images were reconstructed to achieve an effective voxel size of 18 μm. For comparison of bone loss, the distances of the upper part of the bony crest perpendicular to the line of cemental enamel junction of the 1st and 2nd molars of all mice were measured as the d1 site. In addition, the distance from the height of the bony crest perpendicular to the line of cemental enamel junction of the 2nd and 3rd molars of all mice were also measured as the d2 site. The percentage of bone loss in the wild-type group of the vehicle (2 μL of endotoxin-free water) injected into the right side of the upper 1st and 2nd molar interdental areas (WT LPS^-^, d1 site) was designated as 100% for comparisons with other distance measurements of bone loss. The percentage of bone loss in the wild-type group of the vehicle injected into the right side of the upper 2nd and 3rd molar interdental areas (WT LPS^-^, d2 site) was designated as 100% for comparisons with other distance measurements. In addition, 2 μL (20 μg) of purified lipopolysaccharide from *Porphyromonas gingivalis* was injected into the corresponding left side of the upper 1st and 2nd molar interdental areas (WT LPS^+^, d1 site) and the 2nd and 3rd molar interdental areas (WT LPS^+^, d2 site) as the treatment groups.

Similarly, the percentage of bone loss of the right upper jaws between the 1st molar and 2nd molar interdental alveolar areas of vehicle-injected ALDH2*2 knockin mice was designated as the ALDH2 LPS^-^, d1 site. The percentage of bone loss of the right upper jaws between the 2nd and 3rd molar interdental areas of the vehicle-injected ALDH2*2 knockin mice was designated as the ALDH2 LPS^-^, d2 site group. In addition, the percentage of bone loss of the left upper jaws between the 1st and 2nd molars, and between the 2nd and 3rd molar interdental alveolar areas of the ALDH2*2 knockin mice injected with LPS were designated as the ALDH2 LPS^+^, d1 site group and ALDH2 LPS^+^, d2 site group, respectively. Three-dimensional (3D) reconstruction of micro-CT image was used to compare bone loss in the upper jaw of the areas defined above in each animal.

### 2.7. Histological Observation with Hematoxylin and Eosin Staining

After the examination of micro-CT, all samples were demineralized using 15% EDTA for 4 weeks and embedded with paraffin. The samples were sectioned horizontally (5 μm thickness), perpendicular to the long axis of the teeth, and stained with hematoxylin and eosin for histological observation. The sections were screened from the cemental enamel junction to the apical area vertically and the top first section with bony structure. The expression of osteoblasts in the area of interest of each mouse was analyzed and the total number of osteoblasts was counted.

The interdental areas between 1st and 2nd molar and interdental areas between the 2nd and 3rd molar in the right upper jaws and left upper jaws were defined as the region of interest (ROI) areas of each animal. The average total number of osteoblasts in the top section with bony structure in the ROI areas of each mice of the four different groups (WT LPS^+^, WT LPS^-^, ALDH2 LPS^+^, and ALDH2 LPS^-^), were counted three times under a light microscope (Axiovert 200 M; Carl Zeiss AG, White Plains, NY, USA) and quantified. Comparison of the average total number of osteoblasts in the ROI areas were calculated by using ImageJ software and presented as the mean ± standard deviation (SD). Statistical significance was calculated using one-way analysis of variance (ANOVA) followed by post hoc procedure (Fisher’s least significant difference, LSD) (*p* < 0.05 was considered to be significant)

### 2.8. Statistical Analysis

For in vitro measurement of ALDH2 expression, the results were expressed as means ± standard error from at least three separate experiments. The statistical analyses were determined by Student’s t-test, *p* < 0.05 was considered to be statistically significant. For in vitro studies of MTT assay and in vivo studies of the effects of LPS injection and alcohol exposure on periodontal bone loss and the osteoblasts growth, the results were presented as the mean ± standard deviation (SD). Statistical significance was calculated using one-way analysis of variance (ANOVA) followed by post hoc procedure (Fisher’s least significant difference, LSD) (*p* < 0.05 was considered to be significant).

## 3. Results

### 3.1. Expression of ALDH2 in the Wild-Type and ALDH2 Knockdown Human Osteoblasts

**Figure 1:**
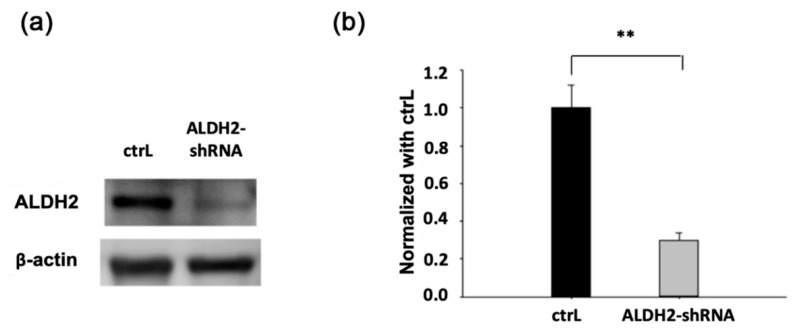
ALDH2 expression in the control transfected human osteoblasts (ctrL) and ALDH2 knockdown transfected osteoblasts (ALDH2-shRNA). (**a**) The expression ALDH2 protein by Western blot analysis; (**b**) quantification of the Western blot showed that in the ALDH2 knockdown osteoclasts, ALDH2 protein level decreased by about 75% (asterisk denotes significant differences, ** *p* < 0.01, *n* = 8).

To evaluate the effect of human osteoblasts that were transfected by lentivirus carrying the human ALDH2-silencing shRNA, ALDH2 protein expression was analyzed by Western blot using the ALDH2 antibody. Compared with the control osteoblasts transfected with an empty lentivirus, ALDH2 protein expression in the ALDH2 knockdown transfected osteoblasts was reduced (Figure 1a, ctrL vs. ALDH2-shRNA). Quantification of the Western blot showed that in the ALDH2 knockdown osteoblasts, the ALDH2 protein level was significantly decreased in only about 25% of the vector-transfected control osteoblasts (Figure 1b, ctrL vs. ALDH2-shRNA).

### 3.2. MTT Assay of the Wild-Type and ALDH2 Knockdown Osteoblasts

**Figure 2:**
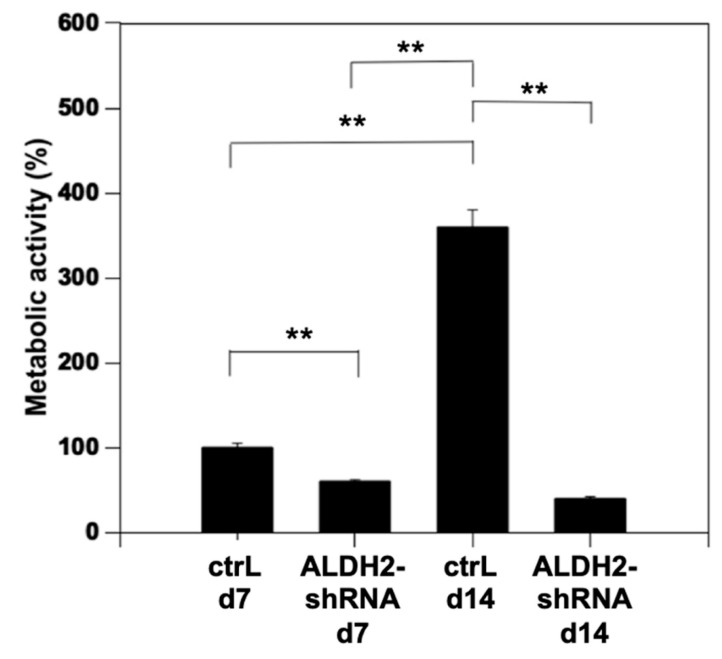
MTT assay of both control (ctrL) and ALDH2 knockdown osteoblasts (ALDH2-shRNA) after 7 days and 14 days of incubation. The metabolic activity of the control and transfected osteoclasts was compared on Days 7 and 14 by the optical density of the formazan formation absorbance using an enzyme-linked immunosorbent assay (asterisk denotes significant differences, ** *p* < 0.01, *n* = 8).

The metabolic activity of the lentivirus transfected osteoblasts was monitored by MTT assay for 14 days after the transfection. On Day 14, metabolic activity in the control vector transfected osteoblasts was significantly higher by 3.6-fold as compared with the osteoblasts that were assayed on Day 7 (Figure 2). However, no increase in metabolic activity was detected in the ALDH2 knockdown osteoblasts (Figure 2). On Day 7, the ALDH2 knockdown osteoblasts already showed a significant reduction in metabolic activity with only about 40% of that of the control group. On Day 14, the metabolic activity of the ALDH2 knockdown osteoblasts was 11.1% of that of the control transfected cells. It showed that knocking down the ALDH2 function could dramatically inhibit the metabolic activities of the osteoblasts and affect the proliferation of the human osteoblasts.

### 3.3. Mineralization Capacity as Measured by Alizarin Red S Staining

**Figure 3:**
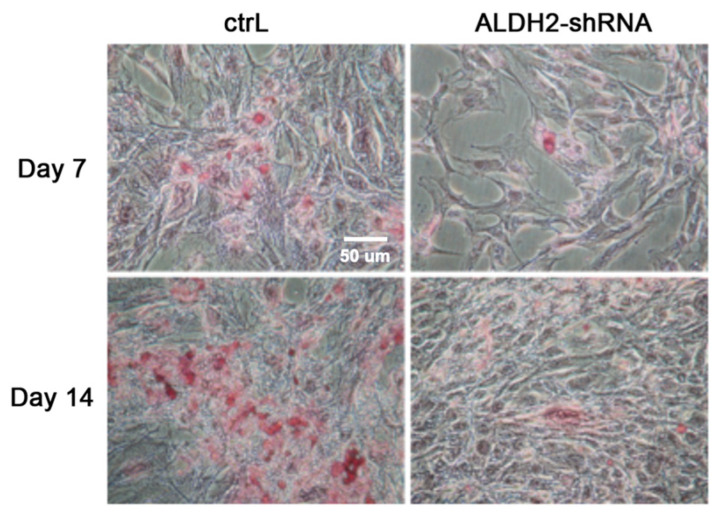
Phase-contrast images of ARS staining of both vector-transfected (ctrL) and ALDH2 knockdown transfected osteoblasts (ALDH2-shRNA) after culturing in differentiation medium for 7 days and 14 days. Pink staining of Alizarin Red S (ARS) indicates the degree of calcification of the osteoblasts. Left panels represent cells transfected by the control vector on Day 7 and Day 14. Right panels represent cells transfected by the ALDH2-shRNA vector on Day 7 and Day 14.

Next, we used a traditional phase-contrast staining method to evaluate the calcification of transfected osteoblasts. Alizarin Red S (ARS) staining was used to quantify the mineralization abilities of the osteoblasts on Day 7 and on Day 14 after transfection using the matrix calcification index, as described by Ratisoontorn et al. [23]. On Day 7, vector-transfected control osteoblasts showed confluence and positive stain of ARS (Figure 3, upper left panel) representing the signal of calcification. A significantly stronger red staining of ARS was further detected on Day 14 of transfection (Figure 3, lower left panel). Importantly, as compared with the control osteoblasts, a weaker ARS staining (14% as compared with the control) was observed in the ALDH2 knockdown transfected osteoblasts on Day 7 (Figure 3, upper right panel). On Day 14, almost no calcification (5% as compared with the control cells on Day 14) was seen in the ALDH2 knockdown transfected osteoblasts (Figure 3, lower right panel). In addition, we also observed that the ALDH2 knockdown transfected osteoblasts grew more slowly and, unlike the control transfected osteoblasts, did not reach confluence on Day 7. These results indicated that the abilities of calcium deposition and proliferation were significantly compromised by ALDH2 inhibition.

### 3.4. Effects of Porphyromonas Gingivalis-Derived LPS Injection on Periodontal Bone Loss in Wild-Type and ALDH2*2 Knockin Mice with Alcohol Intake

**Figure 4:**
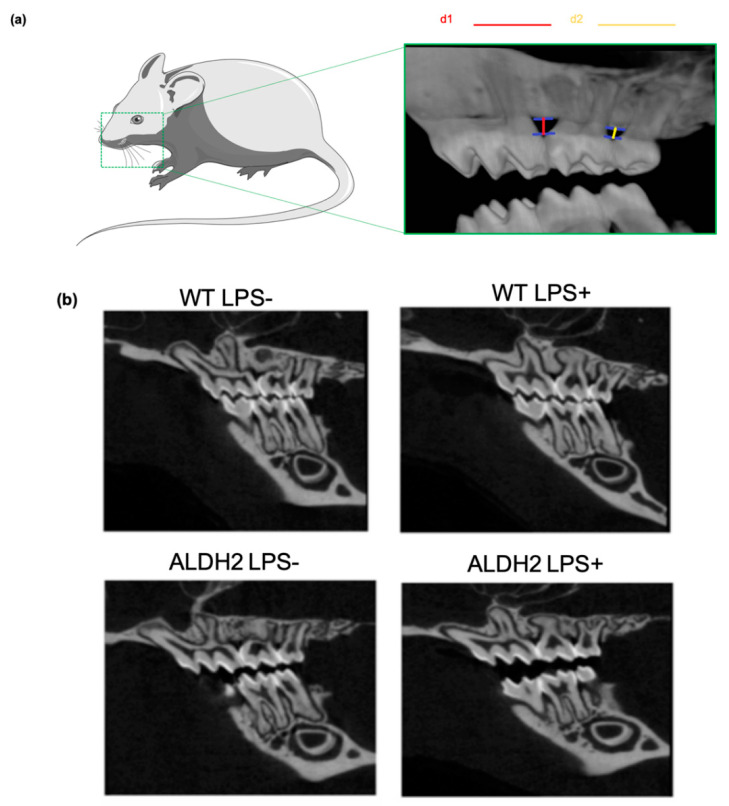
Effects of LPS injection on periodontal bone loss in wild-type and ALDH2*2 knockin mice under alcohol intake. (**a**) 3D reconstruction of micro-CT image demonstrated the comparison of bone loss in the upper jaw of the animal, the distance of the upper part of the bony crest perpendicular to the line of cemental enamel junction of the 1st and 2nd molars of all mice was measured as the d1 site (marked by the red line). The distance from the height of the bony crest perpendicular to the line of cemental enamel junction of the 2nd and 3rd molars of all mice was also measured as the d2 site (marked by the yellow line); (**b**) corrected sagittal images of the LPS-injected left upper jaw (LPS^+^) and water-injected right upper jaw (LPS^-^) of the mice. The 1st molar is shown with three cusps, the 2nd molar is shown with two cusps, and the 3rd molar is the smallest one in three upper molars on each side of the upper jaw. The wild-type mice without LPS injection are labeled as (WT LPS^-^), the wild-type mice injected with LPS are labeled as (WT LPS^+^), the ALDH2*2 knockin mice without LPS injection are labeled as (ALDH2 LPS^-^) and the ALDH2*2 knockin mice injected with LPS are labeled as (ALDH2 LPS^+^).

We used a genetic model of ALDH2*2 knockin mice to evaluate the effect of periodontal bone defect in vivo with age-matched wild-type mice as a control. The ALDH2*2 knockin mice mimics the ALDH2*2 single amino acid substitution of ALDH2*2 mutation in humans [20]. We also utilized a well-established protocol of *Porphyromonas gingivalis* LPS-induced dental bone loss by localized injection of purified LPS (20 ug in 2 uL of endotoxin-free water) to mimic the bacterial infection and inflammation of the upper jaw interdental alveolar bone area on the left sides of the region of interest (ROI) areas in all mice. In addition, drinking water with 10% alcohol was provided every day continuously to mimic the effects of chronic alcohol drinking. We compared the percentage of bone loss in the left side of the upper jaws between the 1st and 2nd, and between the 2nd and 3rd molar interdental areas between the wild-type mice and the ALDH2*2 mice injected with 20 μg (2 μL in endotoxin-free water) of purified lipopolysaccharide from *Porphyromonas gingivalis* and the corresponding animal groups injected with the vehicle control (2 μL of endotoxin-free water). Three-dimensional (3D) reconstruction of micro-computed tomography (micro-CT) image was used to compare bone loss in the upper jaw of the animals. Figure 4a shows a 3D reconstruction of micro-CT image. For the comparison of bone loss, the distance of the upper part of the bony crest perpendicular to the line of cemental enamel junction of the 1st and 2nd molars of all mice was measured as the d1 site. The distance from the height of the bony crest perpendicular to the line of cemental enamel junction of the 2nd and 3rd molars of all mice were also measured as the d2 site. Figure 4b shows the represented sagittal images of the upper and lower jaw bones with posterior molars for four groups of mice including the wild-type animal group with LPS injection (WT LPS^+^), the water-injected wild-type group (WT LPS^-^), the ALDH2*2 knockin LPS-injected group (ALDH2 LPS^+^), and the ALDH2*2 knockin water-injected control group (ALDH2 LPS^-^). There are three upper molar teeth on each side of the upper jaw of each mouse. In Figure 4b, the 1st molar is shown with three cusps, the 2nd molar is shown with two cusps, and the 3rd molar is the smallest one of the three upper molars on each side of upper jaw. We analyzed the ROI areas and calculated the d1 and d2 parameters for all mice in each group. The percentage of bone loss in the right side of the upper 1st and 2nd molar interdental areas of the wild-type water-injected control group (WT LPS^-^, d1 site) was calculated as 100%. The distances were measured and calculated in all other groups and compared with the wild-type control group, as shown in Supplementary Materials Figure S1. We found that there were no differences of bone loss in the ROI areas at the d1 and d2 sites of the wild-type group with LPS (WT LPS^+^) or without LPS injection (WT LPS^-^) (Figure 4b and Figure S1). The bone loss in the ROI areas at the d1 and d2 sites of the ALDH2*2 knockin mice without LPS injection (ALDH2 LPS^-^) showed similar bone loss at the d1 and d2 sites as the wild-type mice with LPS (WT LPS^+^) or without LPS injection (WT LPS^-^) (Figure S1). However, the bone loss at the d1 and d2 sites of the ALDH2*2 knockin mice with LPS injection (ALDH2 LPS^+^) showed 30% higher bony loss at the d1 site and 30% higher bony loss at the d2 site than the corresponding sites of the wild-type animals (WT LPS^-^ and WT LPS^+^) and those of the ALDH2*2 knockin mice without LPS injection (ALDH2 LPS^-^) (Figure S1). Since drinking water with alcohol was provided to all animal groups every day for 6 weeks, the results indicated that under the condition with alcohol intake only, there was no difference in bone loss between the wild-type and ALDH2*2 mice without LPS injection. However, in the ALDH2*2 knockin mice, under the condition of alcohol intake and LPS-injection, bony defects were significantly increased by about 30% as compared with those of the wild-type mice (both WT LPS^-^ and WT LPS^+^) and those of the ALDH2*2 knockin mice without LPS injection (ALDH2 LPS^-^) (Figure S1). In summary, based on micro-CT imaging of 3D reconstruction, the degree of bony defects among the four investigated groups was as follows: (ALDH2 LPS^+^) > (ALDH2 LPS^-^) = (WT LPS^+^) = (WT LPS^-^).

To support our findings, a histological analysis of hematoxylin and eosin (H&E) stained paraffin sections of the upper jaws of all mice was also carried out. We counted the total numbers of osteoblasts in the region of interest (ROI) areas of all specimens from both sides of the upper jaws of the four different animal groups.

**Figure 5:**
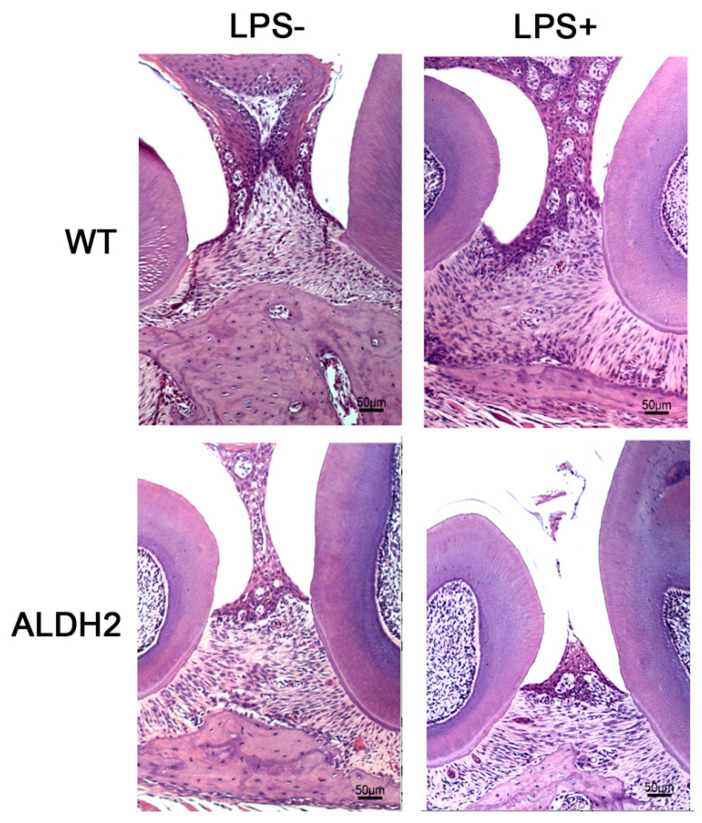
Effects of LPS injection on the osteoblast growth in the periodontal tissue of wild-type mice and ALDH2*2 knockin mice under alcohol intake. Representative H&E images of wild-type (WT) and ALDH2*2 knockin mice without the treatment of LPS injection (left panels) and with the treatment of LPS injection (right panels).

Figure 5 shows the H&E staining of the horizontal sections of the ROI areas from the wild-type mice with or without LPS injection (WT LPS^-^ and WT LPS^+^, Figure 5 top panels), and the ALDH2*2 knockin mice with or without LPS injection (ALDH2 LPS^-^ and ALDH2 LPS^+^, Figure 5 bottom panels). We found that the bony structure in the ALDH2*2 knockin mice with the LPS treatment (ALDH2 LPS^+^) was significantly diminished as compared with the ALDH2*2 mice without LPS injection (ALDH2 LPS^-^, Figure 5 bottom panels). The bony structure in the wild-type mice with LPS injection (WT LPS^+^) was also reduced as compared with the group without LPS injection (WT LPS^-^, Figure 5 top panels). Quantitation of osteoblast numbers showed that in the wild-type mice, the total number of the osteoblasts in the LPS-treated group (WT LPS^+^) was reduced by 17% as compared with the non-treated group (WT LPS^-^, Figure S2, *p* < 0.05). In the ALDH2*2 knockin mice, the total number of the osteoblasts in the LPS treated group (ALDH2 LPS^+^) was reduced by 21% as compared with the untreated group (ALDH2 LPS^-^, Figure S2, *p* < 0.05). Comparing the effects of LPS injection in the wild-type mice and ALDH2*2 knockin mice, the total number of osteoblasts in the ALDH2*2 knockin mice (ALDH2 LPS^+^) was only about 80% of that of the wild-type mice (WT LPS^+^, Figure S2, *p* < 0.05) indicating that the ALDH2*2 mutation rendered the mice more susceptible to the *Porphyromonas gingivalis* LPS toxin. Comparing the wild-type mice and the ALDH2*2 knockin mice without LPS injection, we found that the total number of osteoblasts in the ALDH2*2 knockin mice (ALDH2 LPS^-^) was only about 72% as compared with that of the wild-type mice (WT LPS^-^). This indicated that alcohol intake alone, without LPS injection, was able to inhibit bone regeneration potential with the ALDH2*2 genotype, although we did not observe this difference using the technique of micro-CT imaging (Figure 4). As expected, the greatest effect on the inhibition of osteoblast regeneration was found in the group of ALDH2*2 knockin mice with LPS injection (ALDH2 LPS^+^). In this group, the number of osteoblasts was reduced by 33.3% as compared with the wild-type mice that did not receive the treatment of LPS toxin (WT LPS^-^, Figure S2, *p* < 0.05). These findings indicated that under alcohol intake, the ALDH2 mutation by itself may reduce the growth of the osteoblasts and injection of *Porphyromonas gingivalis* LPS toxin further exacerbated the growth of osteoblasts in the ALDH2*2 knockin mice.

## 4. Discussion

A previous study has revealed a significant association between the ALDH2 Glu504Lys (or ALDH2*2) polymorphism and osteoporosis [24]. The presence of the mutant ALDH2 Lys (or ALDH2*2) allele, particularly in women, has been found to be adversely associated with the risk of osteoporosis [24]. We hypothesized that the ALDH2 mutation had a negative effect on osteoblast activity and dental bone regeneration. Both in vitro and in vivo studies were performed and reported in this study. For the in vitro study, suppression of ALDH2 in human osteoblasts was achieved by transfecting the cells with a lentivirus carrying the ALDH2 shRNA (MOI = 10). We found that the expression of ALDH2 proteins in the ALDH2 knockdown osteoblasts was only 30% of the vehicle transfected control osteoblasts. Fourteen days after the transfection, only 11.1% of the metabolic activity remained in the ALDH2 knockdown osteoblasts as compared with the vehicle transfected control osteoblasts as measured by MTT assay. We also showed that the mineralization capability of ALDH2 knockdown osteoblasts was only about 5% of the untransfected osteoblasts after 14 days in culture. These results indicated that the ALDH2 function played a key role for maintaining the metabolic activity and bone regeneration potential of osteoblasts. A previous study also reported that the mutation of ALDH2 contributed to the inhibition of osteoblast differentiation [25]. Hiroko et al. reported that osteoblasts from an ALDH2*2 overexpressing knockin mice lost their osteogenesis abilities and easily caused osteoporosis [26]. Moreover, Kayoko et al. indicated that the ALDH2*2 knockin mice showed reduced osteoblastic differentiation in bone marrow of trabecular bone under stress [27,28,29]. These studies supported our results that inhibition of ALDH2 had a significant effect on reducing the capacity of mineralization in human osteoblasts.

For the in vivo study, we compared dental bony defects in wild-type and ALDH2*2 knockin mice with or without the challenge of purified LPS toxin derived from *Porphyromonas gingivalis* lipopolysaccharides for 6 weeks under the exposure of 10% alcohol in drinking water. Excess alcohol drinking has been shown to directly cause systemic diseases. Many studies have also indicated that alcohol consumption is a risk factor for periodontitis [30,31]. Alcohol can cause injury to neutrophils and can increase the growth and penetration of bacteria such as *Porphyromonas gingivalis* [32,33]. These bacteria could then induce an inflammation response and promote dental bone loss. Since ALDH2 plays a crucial role in the clearance of toxic acetaldehyde derived from alcohol drinking and the ALDH2 mutation may cause severe bony defects, therefore, we provided drinking water with 10% alcohol to all animals in this study. *Porphyromonas gingivalis* is a well-known periodontal pathogen and is one of the predominant species in the gingival pockets of patients with advanced and severe periodontal disease [22,23]. It is mostly found in the deep periodontal pockets and active sites [34,35]. LPS derived from *Porphyromonas gingivalis* has been demonstrated to be able to cause bone resorption and inhibit bone formation as a culprit of chronic periodontitis [36,37]. In this study, we used purified LPS from *Porphyromonas gingivalis* to create periodontal inflammation and bony defects. Previous studies have shown that LPS from *Porphyromonas gingivalis* prevented apoptosis of HL60-derived neutrophils and the signaling of *P. gingivalis* through toll-like receptor 2 (TLR2) and may account for the inhibitory effect of *P. gingivalis* LPS on apoptosis, thus, provided a mechanism for the development of destructive periodontal disease [38,39]. In addition, Suh et al. showed that injection of *P. gingivalis* LPS caused a more severe periodontitis in mice [40]. Zambrano et al. also reported that *P. gingivalis* LPS could activate the p38 MAPK and NF-κB signaling pathways and induce periodontitis and gingival bone resorption [28]. These studies strongly imply that *P. gingivalis* LPS is a strong inducer of oral bone resorption and periodontal diseases. In our study, we found that *P. gingivalis* LPS-injected ALDH2*2 knockin mice had a significantly higher percentage of bone loss as compared with the LPS-injected wild-type mice and both ALDH2*2 knockin and wild-type mice without LPS injection based on micro-CT scan (Figure S1).

By using histological staining, we found that both the ALDH2*2 genotype and LPS injection incurred a greater loss of osteoblasts in the ROI areas of the four groups we investigated. The combination of ALDH2*2 genotype and LPS treatment had the worst outcome of osteoblast loss among the four groups with the following order: (ALDH2 LPS^+^) > (ALDH2 LPS^-^) > (WT LPS^+^) > (WT LPS^-^) (Figure 5 and Figure S2). We noted that by micro-CT imaging, only the LPS-injected ALDH2*2 knockin mice showed significant bony defects as compared with the other three groups. Micro-CT scanning only reveals the degree of bony defects and not the full potential of bone regeneration reflected by the total number of osteoblasts, therefore, we attributed this difference to the fact that the assessment was less than optimal using micro-CT scanning for detection. Our results also showed that alcohol alone is a risk factor for bony defects in ALDH2*2 genotype, since we observed a reduced number of osteoblasts in the ALDH2*2 knockin mice with LPS injection as compared with the wild-type mice. There is accumulating evidence that the ALDH2*2 inactive mutation is highly related to alcohol-induced systemic diseases such as cardiovascular diseases, neurogenerative diseases, and upper digestive tract cancers including oral cancer [41,42]. The ALDH2*2 mutation is the most common and limited to the East Asian ethnic group with nearly 560 million carriers [43]. Nishida et al. analyzed the association of ALDH2 genotype and body mass index in periodontitis [18]. However, few studies have investigated the relationships among ALDH2 genotype, dental bone loss, and alcohol exposure directly. Therefore, this is the first study on the use of both the ALDH2 gene silencing method and the ALDH2*2 knockin animal model to investigate the relationship between dental bone loss and ALDH2 mutation under the influence of alcohol consumption and LPS toxin from oral bacterium *P. gingivalis*, the two well-known risk factors of periodontitis.

We believe that these results are important for periodontitis and warrant confirmation by epidemiological and clinical studies in human populations where ALDH2*2 mutation, alcohol drinking, and poor oral hygiene are common. Such confirmation would benefit future development of precision dental care and treatment or prevention of dental bone loss among East Asians with the ALDH2*2 genotype.

## 5. Conclusions

In this study, we investigated the roles of ALDH2, alcohol, and *Porphyromonas gingivalis* LPS-induced toxicity in dental bone loss. The ALDH2 function was suppressed by silencing its gene activity in human osteoblasts. After transfecting cells with a lentivirus vector expressing ALDH2 shRNA, ALDH2 protein level was reduced by 80% as compared with the non-transfected osteoblasts. The ALDH2 knockdown osteoblasts lost their proliferation capability and, concomitantly, had reduced mineralization potential. Using a genetic animal model of ALDH2, we compared dental bone loss in wild-type and ALDH2*2 knockin mice with the injection of purified *Porphyromonas gingivalis* LPS and the administration of 10% alcohol in drinking water for 6 weeks. The micro-CT scan results indicated that dental bone loss was significantly more serious in the ALDH2*2 knockin mice injected with LPS. We also found that alcohol alone is a risk factor for bony defects in animals carrying the ALDH2*2 mutation based on the histology of stained bony samples, and the ALDH2 mutation together with LPS injection exacerbated the extend bony defects. These results suggest that the genetic factor of ALDH2 mutation interacts with lifestyle factors, such as alcohol consumption and poor oral hygiene such as the oral toxicity of bacterial-induced *Porphyromonas gingivalis* LPS, and therefore plays a crucial role in the pathology of dental bone loss in periodontal disease. Our findings may have important clinical implications for the dental health of 560 million East Asians carrying the ALDH2*2 mutation.

## Supplementary Materials

The following are available online at https://www.mdpi.com/article/10.3390/biom11050651/s1, Figure S1: Comparison of percentage of periodontal bone loss in each group mice. The percentage of bone loss in the control group non LPS injected right side upper 1st molar and 2nd molar interdental area (WT LPS^-^, d1) was calculated as 100% and the other distance measured were compared with it, Figure S2: Quantitative analysis from H&E images from Figure 5 of LPS injection on the osteoblasts growth in the periodontal tissue of wild type mice and ALDH2*2 knockin mice under alcohol intake.
