# Supplementary material for: The Role of Alcohol, LPS Toxicity, and ALDH2 in Dental Bony Defects
Source: Biomolecules. 2021 Apr 28;11(5):651. doi: 10.3390/biom11050651 (PMC8145216; doi:10.3390/biom11050651)
Supplement: Supplementary file 1 [file biomolecules-11-00651-s001.zip › biomolecules-1162182-supplementary.pdf]

Supporting Information

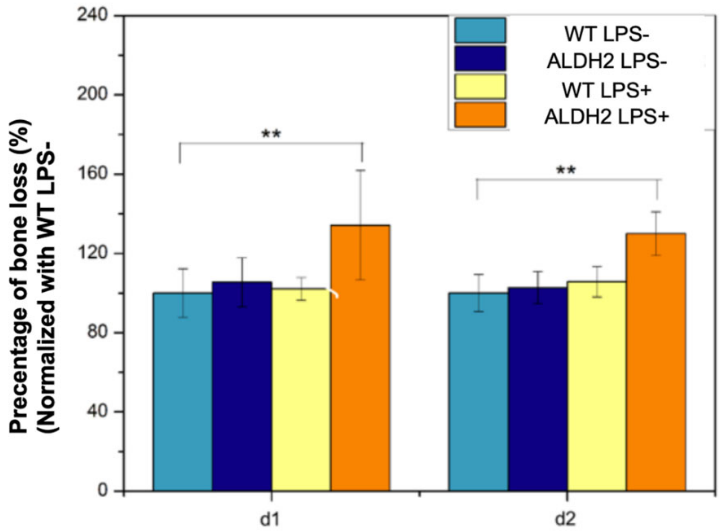

Figure S1. Comparison of percentage of periodontal bone loss in each group mice. The percentage of bone loss in the control group non LPS injected right side upper 1st molar and 2nd molar interdental area (WT LPS-, d1) was calculated as 100% and the other distance measured were compared with it. (*All data compared with WT LPS-. Statistical analysis was performed by One-way ANOVA and post hoc analysis. Asterisk denotes significant differences **$P<0.01$, $n=4$, standard error ± 10%).

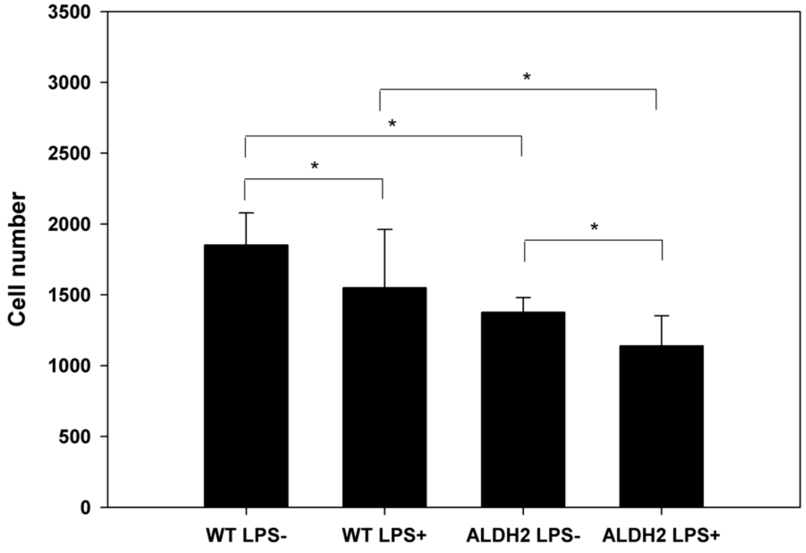

Figure S2. Quantitative analysis from H&E images from Figure 5 of LPS injection on the osteoblasts growth in the periodontal tissue of wild type mice and ALDH2*2 knockin mice under alcohol intake. *Asterisk denotes significant differences *$p< 0.05$, $n= 4$, standard error ± 250).
